# Reaction Products of β-Aminopropioamidoximes Nitrobenzenesulfochlorination: Linear and Rearranged to Spiropyrazolinium Salts with Antidiabetic Activity

**DOI:** 10.3390/molecules27072181

**Published:** 2022-03-28

**Authors:** Lyudmila Kayukova, Anna Vologzhanina, Pavel Dorovatovskii, Gulnur Baitursynova, Elmira Yergaliyeva, Ayazhan Kurmangaliyeva, Zarina Shulgau, Sergazy Adekenov, Zhanar Shaimerdenova, Kydymolla Akatan

**Affiliations:** 1 JSC A. B. Bekturov Institute of Chemical Sciences, 106 Shokan Ualikhanov St., Almaty 050010, Kazakhstan; guni-27@mail.ru (G.B.); erg_el@mail.ru (E.Y.); ayazhankb98@mail.ru (A.K.); 2 A. N. Nesmeyanov Institute of Organoelement Compounds, Russian Academy of Sciences, 28 Vavilova St., B-334, 119991 Moscow, Russia; 3 NRC “Kurchatov Institute”, 1, Akademika Kurchatova pl., 123182 Moscow, Russia; paulgemini@mail.ru; 4 RSE National Center for Biotechnology, Science Committee of ME&S RK, 13/5 Kurgalzhinskoe Highway, Nur-Sultan 010000, Kazakhstan; shulgau@biocenter.kz; 5 JSC International Research and Production Holding Phytochemistry, 4 M. Gazalieva St., Karaganda 100009, Kazakhstan; info@phyto.kz (S.A.); zh.shaimerdenova@phyto.kz (Z.S.); 6 National Scientific Laboratory, S. Amanzholov East Kazakhstan State University, 18/1 Amurskaya St., Ust-Kamenogorsk 070002, Kazakhstan; ahnur.hj@mail.ru

**Keywords:** β-aminopropioamidoximes, nitrobezenesulfochlorination, spiropyrazolinium salts, X-ray diffraction

## Abstract

Nitrobenzenesulfochlorination of β-aminopropioamidoximes leads to a set of products depending on the structure of the initial interacting substances and reaction conditions. Amidoximes, functionalized at the terminal C atom with six-membered *N*-heterocycles (piperidine, morpholine, thiomorpholine and phenylpiperazine), as a result of the spontaneous intramolecular heterocyclization of the intermediate reaction product of an S_N_2 substitution of a hydrogen atom in the oxime group of the amidoxime fragment by a nitrobenzenesulfonyl group, produce spiropyrazolinium *ortho-* or *para*-nitrobenzenesulfonates. An exception is *ortho*-nitrobenzenesulfochlorination of β-(thiomorpholin-1-yl)propioamidoxime, which is regioselective at room temperature, producing two spiropyrazolinium salts (*ortho*-nitrobezenesulfonate and chloride), and regiospecific at the boiling point of the solvent, when only chloride is formed. The *para*-Nitrobezenesulfochlorination of β-(benzimidazol-1-yl)propioamidoxime, due to the reduced nucleophilicity of the aromatic β-amine nitrogen atom, is regiospecific at both temperatures, and produces the *O*-*para*-nitrobenzenesulfochlorination product. The antidiabetic screening of the new nitrobezenesulfochlorination amidoximes found promising samples with in vitro α-glucosidase activity higher than the reference drug acarbose. ^1^H-NMR spectroscopy and X-ray analysis revealed the slow inversion of six-membered heterocycles, and experimentally confirmed the presence of an unfavorable stereoisomer with an axial N–N bond in the pyrazolinium heterocycle.

## 1. Introduction

Heterocycles with potential bioactive properties are of great interest, first of all, for medical chemists working in the field of heterocyclic compounds synthesis. Among the new drugs approved by the FDA in 2021, almost 50% are substances with nitrogen-containing heterocycles [1]. Pyrazoline derivatives, as prominent representatives of nitrogen-containing heterocycles, became the subject of a report on the world market of diphenylpyrazolines from Market Strides (global aggregator and publisher of market intelligence research reports). The main segments of the diphenylpyrazoline market are divided into pharmaceutical and industrial, and address textiles, detergents, paper production, cosmetics, plastics, ceramics, and medicines [2].

**Figure 1:**
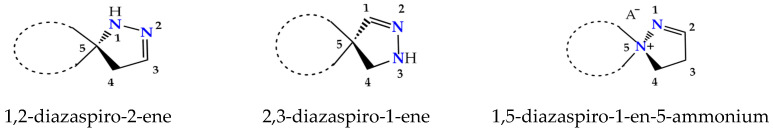
Structural isomers of spiropyrazolines.

Spiropyrazolines, also termed as spirocyclic hydrazine moiety, are rigid asymmetric heterocyclic structures with a chirality axis and with possible centers of chirality C-5 carbon atoms in the most studied 1,2-diazospiro- and 2,3-diazospiropyrazolines or the ammonium nitrogen atom N(+)-5 in 1,5-diazospiropyrazolinium systems (Figure 1).Thus, these compounds exhibit great synthetic potential due to an enantiomeric advantage, regioisomeric composition, reactivity, and tautomeric transformations. Essentially, 1,2- and 2,3-spiro-1-pyrazolines exist as spiro-2-pyrazolines (∆2 isomers) [3], although, in solutions, they exhibit an equilibrium of the imine and enamine forms [4]. As we know, the spiropyrazolinium salts with a 1,5-diazaspiro-1-en-5-ium fragment that we obtained via the hydrolysis of 3-(β-heteroamino)ethyl-5-aryl-1,2,4-oxadiazoles and the arylsulfochlorination of β-aminopropioamidoximes exist, under standard conditions, as the ∆2 isomer [5,6,7,8]. The thermodynamic advantage of possible tautomers of the key pyrazoline moiety was estimated; it turns out that, in the case of pyrazolines, the ∆2 isomer is much more stable than the others [9].

Two comprehensive reviews on functionalized spiropyrazolines were published in 2013 and 2019 [10,11].

The most common methods for the construction of the 1,2- and 2,3-spiro-1-pyrazolines involve the formation of a new ring on an existing carbo- or heterocycle, having exocyclic C=C double bonds. The essential steps in the formation of spiropyrazoline systems in these cases are 1,3-cycloaddition reactions of nitrogen-containing molecules (nitrilimines [12,13,14,15] and diazoalkanes [16,17]) to double bonds, or a condensation reaction of substituted chalcones with hydrazine or its derivatives in an acidic or alkaline medium [18,19].

**Scheme 1:**
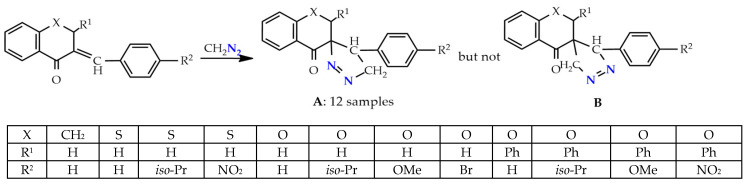
1,3-Dipolar cycloaddition of diazomethane to 3-arylideneflavanones.

There is information about the regioselective and regiospecific syntheses of spiropyrazolines. The synthesis of only one stereoisomer of spiro-1-pyrazolines **A**—∆2 1,2-diazospiropyrazolines obtained by the 1,3-dipolar cycloaddition of diazomethane to 3-arylideneflavanones/chromanones proceeds regioselectively in one step (Scheme 1). The alternative structures **B** were rejected based on the ^1^H NMR and XRD, data as well as the DFT calculations [20,21].

**Scheme 2:**
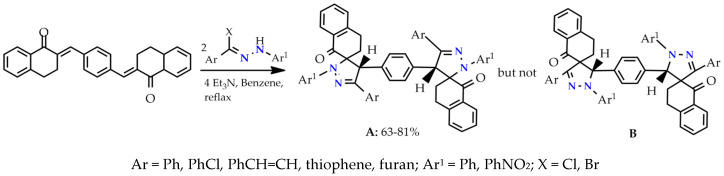
Cycloaddition of bis-exocyclic olefins with nitrile imines.

Symmetrical spiropyrazoline systems are formed by the 1,3-dipolar cycloaddition reaction of (2*E*,2′*E*)-2,2′-(1,4-phenylene bis(methanylylidene)) bis(3,4-dihydronaphthalen-1(2*H*)-one) with a hydrazinoyl halides (nitrile imines). The reaction proceeds regioselectively and only one of two possible spiropyrazoline regioisomers (the 1,2-diazaspiropyrazoline, but not 2,3-diazaspiropyrazoline) is formed. The best explanation for the regioselectivity of the reaction and the impossibility of the formation of the 2,3-diazospiropyrazolines adduct is made using the molecular orbital theory in the interaction of HOMO and LUMO reagents (Scheme 2) [22].

**Scheme 3:**
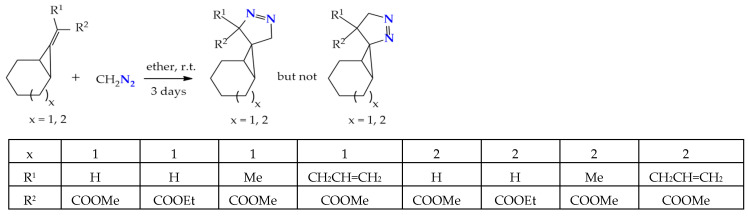
Formation of 2,3-diazaspiro-2-ene isomers in the course of the regiospecific addition of diazomethane to alkylidenecyclopropanes.

1,3-Dipolar cycloaddition of diazomethane to a double bond activated by an electron-withdrawing group in alkylidenecyclopropanes produces 2,3-diazaspiropyrazolines in excellent amounts (95–99%). The determination of the stereochemistry of spiropyrazolines was carried out by NMR spectroscopy, including NOE experiments, which indicated that the methylene protons of the 2,3-diazaspiropyrazoline ring have NOE effects on cyclopropane protons, and this is possible when the methylene group is in the exo-position (Scheme 3) [23]. 

**Scheme 4:**
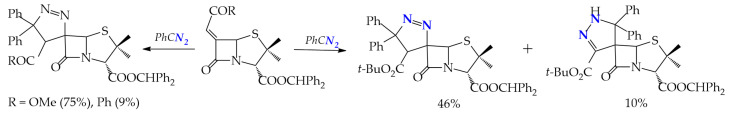
1,3-Dipolar addition of diphenyldiazomethane to 6-alkylidenepenicillanates (reaction conditions: DCM, 30 °C, 24 h).

At a 1,3-dipolar addition of diphenyldiazomethane to 6-alkylidenepenicillanates, examples of regioselective and regiospecific syntheses with the formation of 1,2- and 2,3-spiropyrazoline systems are observed. Regio- and stereospecificity is observed in the interaction of diphenyldiazomethane with penicylates having Ph and COMe alkylidene substituents R to form 1,2-spiropyrazolines: (4′*S*,6*S*)-3-benzhydryl 4′-methoxycarbonyl- and (4′*S*,6*S*)-3-benzhydryl 4′-benzoyl-5′,5′-diphenyl-4′,5′-dihydrospiro[penicillanate-6,3′-(3*H*-pyrazole)]-3-carboxylates up to 75%. The change of the alkylidene substituent to CO_2_*t*-Bu leads to a stereoselective, but regioisomeric, mixture of 1,2- and 2,3-spiropyrazolines: (4′*S*,6*S*)-3-benzhydryl 4′-*tert*-butoxycarbonyl-5′,5′-diphenyl-4′,5′-dihydrospiro[penicillanate-6,3′-(3*H*-pyrazole)]-3-carboxylate and (6*R*)-3-benzhydryl 3′-acetyl-1′,5′-dihydrospiro[penicillanate-6,4′-(4*H*-pyrazole)]-3-carboxylate in a ratio of 46 and 10% (Scheme 4) [24]. 

**Scheme 5:**
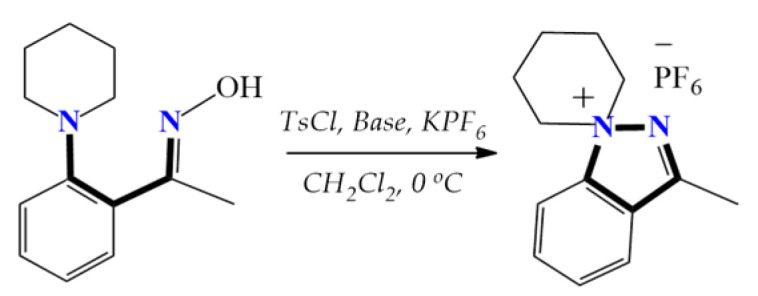
The attainment of stable 1,1-disubstituted indazolium hexafluorophosphates.

Except for our own studies, no examples of 1,5-diazaspiro-1-en-5-ium spiropyrazolinium salts can be found in the literature. However, stable indazole derivatives, 1,1-disubstituted indazolium hexafluorophosphates with a nitrogen atom at the head of the bridge, were obtained utilizing the tosylation reaction. This transformation is similar to that observed in our previous studies, and includes intramolecular neighboring group participation, finally leading to strong bonding, which follows the S_N_2 reaction mechanism (Scheme 5) [25].

Previously, we obtained new spiropyrazolium compounds via the hydrolysis of 3-(β-heteroamino)ethyl-5-aryl-1,2,4-oxadiazoles [5,6], by the arylsulfochlorination (Aryl: *para*-XC_6_H_4_SO_2_Cl; X=CH_3_O, CH_3_, H, Br, Cl, NO_2_) of β-(morpholin-1-yl)aminopropioamidoxime [7] and by the tosylation of β-aminopropioamidoximes (β-amino group: piperidin-1-yl, morpholine-1-yl, thiomorpholin-1-yl, 4-phenylpiperazin-1-yl, benzimidazol-1-yl) at r.t. [8]. In the present study, we are interested in the regioselectivity of β-aminopropioamidoximes *ortho*-, *para*-nitrobenzenesulphochlorination at reaction conditions (chloroform (CHCl_3_), diisopropylamine (DIPEA), room temperature and 70 °C). Depending on the reactant’s structure and reaction temperature, 2-amino-1,5-diazathiospiro[4.5]-dec-1-ene-5-ammonium nitrobenzenesulfonates and chloride and the product of *para*-nitrobenzenesulfochlorination, on the oxygen atom of β-(benzimidazol-1-yl)propioamidoxime were obtained. Among them, samples with high in vitro antidiabetic activity were found. The significance of the present work is the establishment of the ambiguity of amidoximes sulfochlorination and the possibility of obtaining a wide range of potentially biologically active products.

## 2. Results and Discussion

### 2.1. Synthesis and Spectra

The interaction of β-aminopropioamidoximes (**1**–**5**) with *ortho*-, *para*-nitrobenzenesulfochlorides in CHCl_3_ was carried out at room temperature and by heating the reaction mixture to the solvent boiling point. A change in the electronic properties of the sulfochlorinating agent, the transition from tosyl chloride to *ortho*-, *para*-nitrobenzenesulfochlores lead to an increase in the reaction time at room temperature from 15–20 h in the case of tosylation [4] to 38–120 h for *para*-benzenesulfochlorination, and up to 25–104 h for *ortho*-benzenesulfochlorination.

**Scheme 6:**
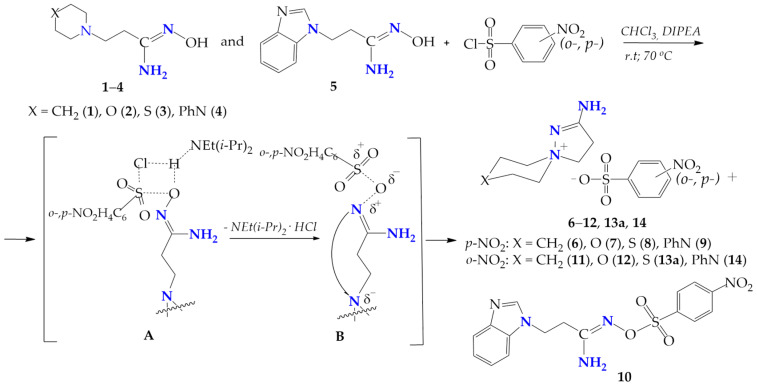
Nitrobenzenesulfochlorination of β-aminopropioamidoximes.

The heating of the reaction mixture at the CHCl_3_ boiling point reduces the reaction time to 19–36 h, and to 24 h for *para*- and *ortho*-nitrobenzenesulfochlorination, respectively. The completion of β-aminopropioamidoximes arylsulfochlorination was confirmed by the physicochemical data (TLC, elemental analysis, m.p.), and the IR and NMR (^1^H and ^13^C) spectra and X-ray data of the isolated products (**6**–**14**). On the basis of physicochemical and spectral data, it was concluded that, in the case of the nitrobenzenesulfochlorination of β-aminopropioamidoximes with six-membered heterocycles in the β-position (**1**–**4**), the main products were the nitrobenzenesulfonates of spiropyrazoline compounds **6**–**12**, **13a**, and **14**; however, when the substrate was β-(benzimidazol-1-yl)propioamidoxime (**5**), only the product of *para*-nitrobenzensulfochlorination at the oxygen atom of the amidoxime fragment **10** was isolated from the reaction mixture (Scheme 6).

It is assumed that, in the case of the nitrobenzenesulfochlorination of β-aminopropioamidoximes **1**–**4**, the *O*-nitrobenzenesulphonates of β-propioamidoximes formed as intermediate **B**, due to the thermodynamic advantage, rearranging into spiropyrazoline nitrobenzenesulphonates (**6**–**12**, **13a**, **14**). Only in the case of the benzimidazole derivative, the intermediate **B** remains stable and produces *O*-*para*-nitrobenzenesulphonate **10**.

**Figure 2:**
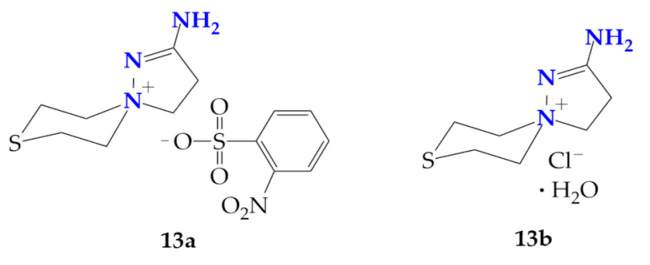
Salts of spiropyrazolinium compounds **13a** and **13b** isolated at the *ortho*-nitrobenzenesufochlorination of β-(thiomorpholine-1-yl)propioamidoxime.

In addition, the *ortho*-nitrobenzenesulfochlorination of β-(thiomorpholine-1-yl)propioamidoxime (**3**) has the following features: at room temperature, a mixture of 2-aminospiropyrazolylammonium *ortho*-nitrobenzenesulfonate and chloride (**13a** and **13b**) were obtained, and carrying out the reaction at 70 °C only produces chloride **13b** (Figure 2). Compound **13b** was previously characterized by us [26,27].

Evidently, in this case, the *para*-nitrophenylsulfonate anion in the primarily formed 2-amino-1,5-diazaspiro[4.5]dec-1-en-5-ium *para*-nitrophenylsulfonate (**13a**) was exchanged for the chloride anion from DIPEA hydrochloride, releasing *para*-nitrobenzenesulfonic acid and DIPEA.

The stoichiometry of the reaction does not provide for the formation of a hydrate. We believe that the preparation of hydrate **13b** can be explained by the prolonged contact of the mother liquor during the preparation of the single crystals of the *ortho*-nitrophenylsulfochlorination product of amidoxime **3** with atmospheric moisture.

When establishing the structure of nitrobenzenesulfochlorination products, a difference was noted in the values of the mobility index *R*_f_ for the products produced by the nitrobenzenesulfochlorination of amidoximes with six-membered nitrogenous heterocycles in the β-position (**6**–**9**, **11**–**14**) and for the *para*-nitrobenzenesulfoderivative of β-(benzimidazol-1-yl)propioamidoxime (**10**) (*R*_f_ 0.01–0.12 and 0.75). Compounds **7** and **13b** were previously described in [7] and [26,27], respectively (Table 1).

In the IR spectra of compounds **6**–**12**, **13a**, and **14**, there are two pairs of bands related to the characteristic stretching vibrations of a strong intensity of the NO_2_ and SO_2_ groups at 1515–1549 (as) cm^−1^ and 1349–1377 (sy) cm^−1^, and 1207–1240 (as) cm^−1^ and 1024–1197 (sy) cm^−1^, respectively, whereas, in the IR spectrum of compound **13b**, there are no bands of stretching vibrations for the bonds of the NO_2_ and SO_2_ groups.

It is interesting that, in the ^1^H-NMR spectra of compounds **6**–**9**, **13a**, and **13b**, it was possible to fix the diastereotopic nature of the geminal protons of the methylene groups located at the ammonium nitrogen atom, which produce pairs of multiplet signals with an intensity of 2 protons at δ: 3.35 m, 3.44 m (**6**); 3.41 m, 3.65 m (**7**); 3.60 m, 3.72 m (**8**); 3.49 m, 3.98 m (**9**); 3.10 m, 3.68 m (**13a**); and 3.10 m, 3.68 m (**13b**).

Similarly, the geminal protons of the methylene groups at the S, O, S, S, and N atoms of the β-heterocycles of compounds **8**, **12**, **13a**, **13b**, and **14** also appear as pairs of proton signals with an intensity of 2 protons at δ: 2.87 m, 3.15 m (**8**); 3.35 m, 3.60 m (**12**); 2.84 m, 3.55 m (**13a**); 2.85 m, 3.55 m (**13b**); and 3.40 m, 3.71 m (**14**). Evidently, the effect of the slow rotation of β-heterocycles with the possibility of fixing the equatorial and axial protons is observed here. In addition, the diastereotopicity of these protons may be associated with the presence of an asymmetry axis inherent in the spiro compounds.

The signals of Csp^3^ and Csp^2^ carbon atoms in the ^13^C-NMR spectra of compounds **6**–**14** are present in the characteristic regions.

We obtained a quantum-chemical confirmation of the advantageousness of the formation of spirocyclic tosylation and *para*-nitrobenzenesulfochlorination products of β-aminopropioamidoximes (**1**–**4**), with negative values of the Gibbs energy of the chemical reaction in the range of −119.99–−163.57 kJ/mol and the disadvantage of the formation of a spirostructure for β-(benzimidazole-1-yl)propioamidoxime (**5**), with positive values of the Gibbs energy for the tosylation and *para*-nitrobenzenesulfochlorination products as 45.87 and 20.02 kJ/mol, respectively [28].

The thermodynamic advantage of the formation of 2-aminospiropyrazolylammonium sulfonates (tosylates, *para*- and *ortho*-nitrobenzenesulfonates) for a number of β-aminopropioamidoximes (**1**, **2**, **4**), in comparison to the formation of chloride hydrates, was identified, except for the case when the initial substrate was β-(thiomorpholine-1-yl)propioamidoxime **3**; in this case, 2-amino-8-thia-1,5-diazaspiro[4.5]dec-1-en-5-ammonium chloride monohydrate (**13b**) is preferred by −8.1, −6.18, −3.08 kJ/mol, respectively [29].

It should be noted that we attempted to react β-(benzimidazol-1-yl)propioamidoxime (**5**) with *ortho*-nitrobenzenesulfonate several times, under the described conditions (CHCl_3_, DIPEA, r.t. and 70 °C), but, each time, a resinous reaction mixture was obtained.

### 2.2. The In Vitro Antidiabetic Screening of 2-Amino-1,5-diazaspiro[4.5]dec-1-en-5-ammonium Nitrobenzenesulphonates and Chloride Hydrate (**6**–**9**, **11**–**14**) and 3-(1H-Benzo[d]imidazol-1-yl)-N′-{[(4-nitrophenyl)sulfonyl]oxy}propanimidamide (**10**)

The α-amylase and α-glucosidase inhibition tests, which hydrolyze starch in postprandial hyperglycemia, are standard tests used in the discovery and development of new antidiabetic drugs. Essentially, the regulation of α-amylase and α-glucosidase biological functions (inhibition) is critical to the treatment regimen. This implies that new drug candidates with a potent inhibition of the α-glucosidase and α-amylase would be valuable to drug discovery and development for diabetes mellitus [30].

The in vitro antidiabetic activity of spiropyrazolilammonium *ortho*-, *para*-nitrobenzenesulfonates and chloride **6**–**9**, **11**–**14**, and the product of *O*-*para*-nitrobenzenesulfochlorination of β-(benzimidazol-1-yl)propioamidoxime (**10**) was assessed via the degree of inhibition of the activity of α-amylase and α-glucosidase by the studied substances, compared to the standard drug acarbose (Table 2). The table shows four series of experiments with different experimental values of the in vitro activity of acarbose in relation to α-amylase and α-glucosidase.

All the tested compounds had an average inhibitory activity against α-amylase, the values of which are less than the activity of acarbose. In regard to α-glucosidase, the products of *para*-nitrobenzenesulfochlorination **8** and **10** had a pronounced inhibitory activity (61.0% and 67.1%). In the same series of experiments, the average inhibitory activity of 36.5% and 48.1% is shown by the compounds **7** and **9**. The reference drug acarbose exhibited the standard inhibitory activity of 58.9% and 50.3% in terms of α-glucosidase and α-amylase, respectively. In two other series of experiments, the tested *ortho*-nitro derivatives (**11**–**14**) did not show activity against α-amylase and α-glucosidase, comparable to the activity of acarbose.

The apparent difference in the antidiabetic activity of the subgroups of compounds **6**–**10** and **11**–**14** can be explained by the difference in their chemical structure. The first subgroup is derivative of *para*-nitrophenylsulfonic acid; the second subgroup is an *ortho*-nitrophenylsulfonic acid derivative. It is likely that binding to the sites of α-amylase and α-glucosidase enzymes responsible for increasing blood sugar is more efficient for *para*-nitrobenzenesulfonic acid derivatives (**6**–**10**), while *ortho*-nitrophenylsulfonic acid derivatives (**10**–**14**) are less efficient for the target reference antidiabetic drug acarbose.

The absence of α-glucosidase activity in representatives of the subgroup of compounds **11**–**14** may be associated with the previously described trend of a general decrease in antidiabetic activity in the series of *ortho*-nitrophenylsulfonic acid derivatives (**10**–**14**), whereas the absence of α-glucosidase activity for compound **6** may be a random variable.

### 2.3. X-ray Diffraction

X-ray diffraction studies of all the reaction products were carried out. They confirmed that, regardless of the synthetic conditions, the nitrobenzenesulfochlorination of β-aminopropioamidoximes containing piperidin-1-yl, morpholine-1-yl and 4-phenylpiperazin-1-yl as β-aminogroup affords spiropyrazolium nitrobenzenesulfonates **6**, **7**, **9** or **11**, **12**, **14**. As a result of the *ortho*-nitrobenzenesulfochlorination of β-thiomorpholin-1-ylpropioamidoxime, nitrophenylsolfonate **13a** and chloride monohydrate **13b** of the corresponding spirocation, similar to the one previously reported [29], can be obtained depending on the reaction conditions. No rearrangement was detected for benzimidazol-1-yl-containing product **10** in accord with the B3LYP/6-31++G(d,p) calculations of the standard Gibbs free energies of reaction [28,29] found for N-substituted aminopyrazoles [31,32]. All salts contain one cation and one anion in the asymmetric unit (Figure S1, ESI). The quality of XRD data allowed us to locate all of the hydrogen atoms on difference Fourier maps, and undoubtedly confirmed that none of the sulfonate groups contained any hydrogen atoms.

**Figure 3:**
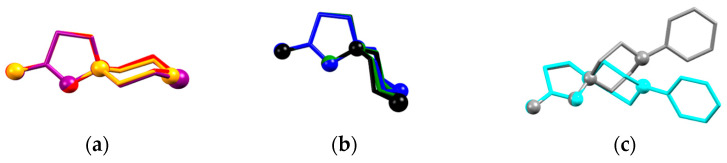
Molecular conformations of the spirocation in (**a**) **7** (orange), **11** (red), **13a** (violet); (**b**) **6** (green), **12** (blue), **14** (black); and (**c**) **9** (grey) and **14** (cyan). The superimposed atoms are N–N=C–C atoms in the 5-membered pyrazole ring.

The molecular structures of these compounds are depicted on Figure S1 (ESI), and the main geometry parameters of the cations and anions are listed in Table 3. The X–CH_2_ bond distances increase and CH_2_–X–CH_2_ angles decrease, passing from O to NPh; CH_2_ and S. Valence angles at the positively charged N1 atom are close to ideal 109.5° values, however only N–CH_2_ bond distances within the 6-membered ring are typical for a single N–C bond. In **10**, a similar N1–CH_2_ bond is equal to 1.458(2) Å, due to the mesomeric effect of the benzimidazole substituent. The N–N bond in these salts varies from 1.460(2) to 1.470(2) Å, which is longer than 1.42–1.43 Å that is found for N-substituted aminopyrazoles [32,33]. Overall the conformations of the cations in **7**, **9**, **11**,**13a**, and **13b** correspond to one conformation on the six-membered ring towards the five-membered ring, while **6**, **8**, **12**, and **14** are the first examples of the inverted chair conformation (Figure 3) of this ring confirmed by means of X-ray diffraction. The difference in the two conformations manifests itself in the ^1^H-NMR spectra (see above), and also through the N1–C1 bond length, which is generally shorter in cations with ‘novel’ conformations. In our opinion, the overall conformation of the molecule and elongation of bond distances in the pyrazole ring can be attributed to the anomeric effect [33] of the (hetero)atom in the six-membered ring. The length of the N2=C bond in all the spirocations is nearly the same as the N2=C bond distance of 1.302(2) in **10**.

**Figure 4:**
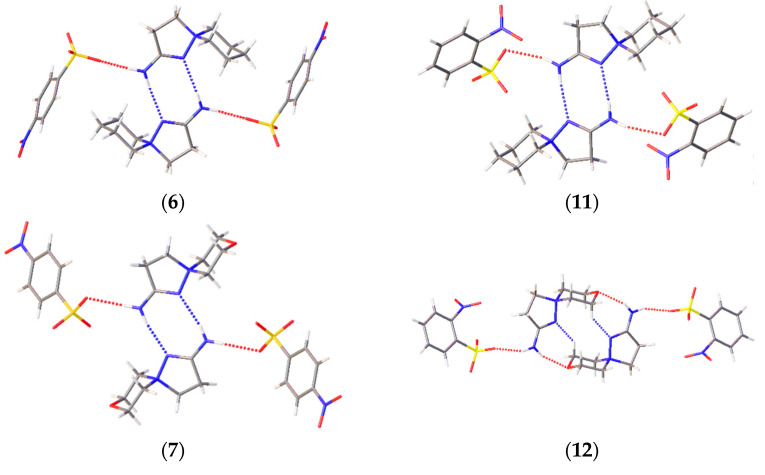

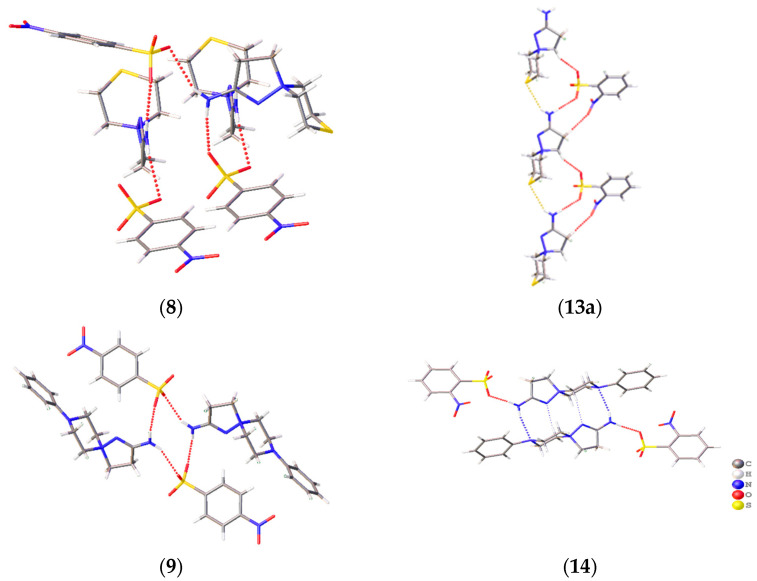
H-bonding patterns in the crystal structures of the studied compounds. H bonds are depicted with dotted lines.

A prominent variation in the biological properties of these compounds can be accounted for the possibility of these cations to realize different conformations and to take part in different types of H bonds [34]. In these solids, the only donor for the H bonds is the amino group, while the sulfonate groups of the anions and heteroatoms of the cations compete to act as acceptors of H bonding. As a result, H-bonded chains are observed in 2-amino-1,5-diazathiospiro[4.5]-dec-1-ene-5-ammonium-containing salts and tetramers in six other salts (Figure 4). The D^3^_3_(9) tetramers in **6**, **7**, and **11** are formed through two N–H…N interactions and two N–H…O ones. Similar D^3^_3_(13) tetramers in **12** and **14** are obtained through a similar N–H…O cation…anion interactions, but the cations are shifted along each other to allow for the bonding with a heteroatom of the six-membered ring. The R^4^_4_(12) cycles in **9** and the H-bonded chains in **8** and **13** are formed by the interactions of the amino group with sulfonate groups of two neighboring molecules. The **G^a^_d_**(**n**) notation of H-bonded architectures is presented in terms of [33], where **G** represents the type of pattern (**C** for chain, **S** for intramolecular hydrogen bonds, **R** for ring, and **D** for finite), **a** is the number of acceptors, **d** is the number of donors, and **n** the number of atoms in the pattern. Note that, despite the similar crystal parameters and space group of **11**–**13** (Table S1, ESI), these compounds cannot be regarded as isostructural, as they realize different packing and intermolecular (including H-bonding) interactions. Crystal packing demonstrates that, for these spirocations, heteroatoms of the six-membered cycle can take part in H bonding, and different conformations can be stabilized by means of intermolecular bonding.

## 3. Materials and Methods

### 3.1. Synthesis

The reagents were purchased from different chemical suppliers and were purified

Before use. FT-IR spectra were obtained on a Thermo Scientific Nicolet 5700 FTIR instrument (Thermo Fisher Scientific, Inc., Waltham, MA, USA) in KBr pellets. The ^1^H- and ^13^C-NMR spectra of compounds **6**–**10** were acquired on a Bruker Avance III 500 MHz NMR spectrometer (Bruker, BioSpin GMBH, Rheinstetten, Germany) and ^1^H- and ^13^C-NMR spectra of compounds **11**–**14** on a Jeol JNM-ECA 500 (Jeol, Tokyo 196-8558, JAPAN), (500 and 126 MHz, respectively).

The signals of the residual undeuterated solvents were used as a reference for the ^1^H-NMR (2.50 ppm) and 13C-NMR (39.5 ppm) spectra. Elemental analysis was carried out on a CE440 elemental analyzer (Exeter Analytical, Inc., Shanghai, China). The melting points were determined in glass capillaries on a PTP(M) apparatus (Khimlabpribor, Klin, Russia). The reaction progress and purity of the obtained products were controlled using Sorbfil (Sorbpolymer, Krasnodar, Russia) TLC plates coated with CTX-1A silica gel, grain size 5–17 μm, containing UV-254 indicator. The eluent for TLC analysis was a mixture of benzene–EtOH, 1:3. The solvents for the synthesis, recrystallization, and TLC analysis (ethanol, 2-PrOH, benzene, DMF, and acetone) were purified according to the standard techniques.

#### 3.1.1. A General Procedure for the Synthesis of 2-Amino-1,5-diazaspiro[4.5]-dec-1-ene-5-ammonium *para*-Nitrobezenesulfonates (**6**–**9**) and 3-(1*H*-Benzo[d]imidazol-1-yl)-*N*′-{[(4-nitrobenzene)sulfonyl]oxy}propanimidamide (**10**)

*The synthesis of β-aminopropioamidoximes4-nitrobenzenesulfochlorinationproducts **6**–****10****(general method).* To a solution of 0.0029 mol of β-aminopropioamidoximes **1**–**5** in 20 mL of CHCl_3_, 0.0029 mol of DIPEA was added. The reaction mixture was cooled to −1 °C, and a solution of 0.0029 mol of 4-nitrobenzenesulfochloride in 2 mL of CHCl_3_ was added dropwise with stirring. The reaction mixture was then allowed to warm to r.t. (or heated to the boiling point (b.p.) of CHCl_3_) and stirred until the completion of the reaction. The progress of the reaction was monitored by TLC. The formed white precipitates of the products **6**–**10** were filtered off and recrystallized from 2-PrOH.

*2-Amino-1,5-diazaspiro[4.5]dec-1-en-5-ammonium 4-nitrobenzenesulfonate* (**6**). The reaction mixture consisting of 0.5 g (0.0029 mol) of compound **1** in 20 mL of CHCl_3_ and 0.38 g (0.0029 mol) of DIPEA was cooled to -1℃. Then, 0.64 g (0.0029 mol) of 4-nitrobenzenesulfochloride was added dropwise. When the reaction mixture was kept at r.t. for 38 h, 0.80 g (77%) of white solid **6** was obtained (when the reaction mixture was kept at CHCl_3_ b.p. for 27 h, 0.48 g (46%) of white solid **6** was obtained); m.p. 151 °C, *R*_f_ 0.12. IR (KBr, cm^−1^): 1665 (C=N), 1607 (C=C), 1232 (SO_2_ as) and 1190 (SO_2_ sy), 1529 (NO_2_ as) and 1352 (NO_2_ sy), 3302 [N(-H)_2_], 2938, 2864, 2815 (C_sp3_−H), 3032, 3257 (C_sp2_−H). ^1^H-NMR (500 MHz, DMSO-*d*_6_): 3.10 (t, J = 7.0 Hz, 2H, α-CH_2_), 3.81 (t, J = 7.0 Hz, 2H, β-CH_2_), 1.55 m, 1.75 m, 1.87 m, [6H, (CH_2_)_3_], 3.35 [m, 2H, N(+)(CH_ax_)_2_] and 3.44 [m, 2H, N(+)(CH_eq_)_2_], 7.23 (s, 2H, NH_2_), 7.83−8.21 (m, 4H, C_(sp2)_H). ^13^C-NMR (126 MHz, DMSO-*d*_6_): 21.0, 21.9, 31.5, 60.7, 64.3 (2C), 126.0 (2C), 128.5 (2C), 138.0 (1C), 145.3 (1C), 168.5. Anal. Calcd for C_14_H_20_N_4_O_5_S (356.40): C, 47.18; H, 5.66. Found: C, 47.36; H, 5.37.

*2-Amino-8-oxa-1,5-diazaspiro[4.5]dec-1-ene-5-ammonium 4-nitrobenzenesulfonate* (**7**).The reaction mixture consisting of 0.5 g (0.0029 mol) of compound **2** in 20 mL of CHCl_3_ and 0.38 g (0.0029 mol) of DIPEA was cooled to −1 °C. Then, 0.64 g (0.0029 mol) of 4-nitrobenzenesulfochloride was added dropwise. When the reaction mixture was kept at r.t. for 120 h, 0.73 g (70%) of white solid **7** was obtained (when the reaction mixture was kept at CHCl_3_ bp for 24 h, 0.77 g (74%) of white solid **7** was obtained); m.p. 187–188 °C, *R*_f_ 0.10. IR (KBr, cm^−1^): 1650 (C=N), 1600 (C=C), 1231 (SO_2_ as) and 1194 (SO_2_ sy), 1520 (NO_2_ as) and 1362 (NO_2_ sy), 3466 [N(-H)_2_], 2967, 2854 (C_sp3_−H), 3236, 3328, 3388 (C_sp2_−H). ^1^H-NMR (500 MHz, DMSO-*d*_6_): 3.13 (t, J = 7.0 Hz, 2H, α-CH_2_), 3.92 (t, J = 7.0 Hz, 2H, β-CH_2_), 3.32 [m, 4H, O(CH_2_)_2_], 3.41 [m, 2H, N(+)(CH_ax_)_2_] and 3.65 [m, 2H, N(+)(CH_eq_)_2_], 7.28 (s, 2H, NH_2_), 7.80−8.19 (m, 4H, C_(sp2)_H). ^13^C-NMR (126 MHz, DMSO-*d*_6_): 31.4, 62.1, 62.4, 63.2, 123.8 (2C), 127.4 (2C), 147.7 (1C), 154.8 (1C), 170.1. Anal. Calcd for C_13_H_18_N_4_O_6_S (358.37): C, 43.57; H, 5.06. Found: C, 43.48; H, 5.13.

*2-Amino-8-thia-1,5-diazaspiro[4.5]dec-1-en-5-ammonium 4-nitrobenzenesulfonate* (**8**). The reaction mixture consisting of 0.55 g (0.0029 mol) of compound **3** in 20 mL of CHCl_3_ and 0.38 g (0.0029 mol) of DIPEA was cooled to −1 °C. Then, 0.64 g (0.0029 mol) of 4-nitrobenzenesulfochloride was added dropwise. When the reaction mixture was kept at r.t. for 84 h, 0.74 g (68%) of white solid **8** was obtained (when the reaction mixture was kept at CHCl_3_ b.p. for 21 h, 0.51 g (47%) of white solid **8** was obtained); m.p. 230 °C, *R*_f_ 0.10. IR (KBr, cm^−1^): 1642 (C=N), 1588 (C=C), 1229 (SO_2_ as) and 1197 (SO_2_ sy), 1519 (NO_2_ as) and 1349 (NO_2_ sy), 3308, 3396 [N(-H)_2_], 2944 (C_sp3_−H), 3105, 3199, 3245 (C_sp2_−H). ^1^H-NMR (500 MHz, DMSO-*d*_6_): 3.11 (t, J = 7.0 Hz, 2H, α-CH_2_), 3.86 (t, J = 7.0 Hz, 2H, β-CH_2_), 2.87 [m, 2H, S(CH_ax_)_2_] and 3.15 [m, 2H, S(CH_eq_)_2_], 3.60 [m, 2H, N(+)(CH_ax_)_2_] and 3.72 [m, 2H, N(+)(CH_eq_)_2_], 7.37 (s, 2H, NH_2_), 7.83−8.20 (m, 4H, C_(sp2)_H). ^13^C-NMR (126 MHz, DMSO-*d*_6_): 23.2, 31.4, 62.5, 64.7, 123.8 (2C), 127.3 (2C), 147.7 (1C), 154.8 (1C), 169.0. Anal. Calcd for C_13_H_18_N_4_O_5_S_2_ (374.44): C, 41.70; H, 4.85. Found: C, 41.59; H, 4.33.

*2-Amino-8-phenyl-1,5,8-triazaspiro[4.5]dec-1-en-5-ammonium 4-nitrobenzenesulfonate* (**9**). The reaction mixture consisting of 0.72 g (0.0029 mol) of compound **4** in 20 mL of CHCl_3_ and 0.38 g (0.0029 mol) of DIPEA was cooled to −1 °C. Then, 0.64 g (0.0029 mol) of 4-nitrobenzenesulfochloride was added dropwise. When the reaction mixture was kept at r.t. for 60 h, 0.88 g (70%) of white solid **9** was obtained (when the reaction mixture was kept at CHCl_3_ b.p. for 19 h, 0.87 g (69%) of white solid **9** was obtained); m.p. 203 °C, *R*_f_ 0.15. IR (KBr, cm^−1^): 1649 (C=N), 1599 (C=C), 1240 (SO_2_ as) and 1189 (SO_2_ sy), 1515 (NO_2_ as) and 1350 (NO_2_ sy), 3421 [N(-H)_2_], 2790, 2880, 2915 (C_sp3_−H), 3118, 3290 (C_sp2_−H). ^1^H-NMR (500 MHz, DMSO-*d*_6_): 3.17 (t, J = 7.0 Hz, 2H, α-CH_2_), 3.95 (t, J = 7.0 Hz, 2H, β-CH_2_), 3.56 [m, 4H, N(CH_2_)_2_], 3.49 [m, 2H, N(+)(CH_ax_)_2_] and 3.98 [m, 2H, N(+)(CH_eq_)_2_], 7.25 (s, 2H, NH_2_), 7.81−8.53 (m, 9H, C_(sp2)_H). ^13^C-NMR (126 MHz, DMSO-*d*_6_): 31.5, 44.5, 61.5, 62.9, 115.3 (2C), 120.4 (1C), 123.8 (2C), 127.4 (2C), 129.5 (2C), 147.7 (1C), 149.9 (1C), 156.7 (1C), 169.0. Anal. Calcd for C_19_H_23_N_5_O_5_S (433.48): C, 52.64; H, 5.35. Found: C, 52.35; H, 5.21.

*3-(1H-benzo[d]imidazol-1-yl)-N′-{[(4-nitrophenyl)sulfonyl]oxy}propanimidamide* (**10**). The reaction mixture consisting of 0.59 g (0.0029 mol) of compound **5** in 20 mL of CHCl_3_ and 0.38 g (0.0029 mol) of DIPEA was cooled to −1 °C. Then, 0.64 g (0.0029 mol) of 4-nitrobenzenesulfochloride was added dropwise. When the reaction mixture was kept at r.t. for 80 h, 0.93 g (82%) of white solid **10** was obtained (when kept at CHCl_3_ b.p. for 36 h, 0.41 g (36%) of white solid **10** was obtained); m.p. 158 °C, *R*_f_ 0.75. IR (KBr, cm^−1^): 1648 (C=N), 1617 (C=C), 1240 (SO_2_ as) and 1187 (SO_2_ sy), 1520 (NO_2_ as) and 1365 (NO_2_ sy), 3417 [N(-H)_2_], 2791, 2920 (C_sp3_−H), 3110, 3237 (C_sp2_−H). ^1^H-NMR (500 MHz, DMSO-*d*_6_): 2.50 (t, J = 7.0 Hz, 2H, α-CH_2_), 4.33 (t, J = 7.0 Hz, 2H, β-CH_2_), 6.50 (s, 2H, NH_2_), 7.78−8.44 (m, 8H, C_(sp2)_H), 8.05 (s, 1H, C_(sp2)_H). ^13^C-NMR (126 MHz, DMSO-*d*_6_): 31.2, 42.9, 110.8 (2C), 119.8 (2C), 121.9 (1C), 122.7 (1C), 128.5 (2C), 130.0 (2C), 133.5 (2C), 134.0 (1C), 143.7 (1C), 144.3 (1C), 144.7 (1C), 160.0. Anal. Calcd for C_16_H_15_N_5_O_5_S (389.39): C, 49.35; H, 3.88. Found: C, 49.28; H, 3.45.

#### 3.1.2. A General Procedure for the Synthesis of 2-Amino-1,5-diazaspiro[4.5]-dec-1-ene-5-ammonium 2-Nitrobezenesulfonate (**11**–**13a**, **14**) and 2-Amino-8-thia-1,5-diazaspiro[4.5]dec-1-en-5-ium Chloride Hydrate (**13b**)

*The synthesis of β-aminopropioamidoximes2-nitrobenzenesulfochlorinationproducts***6**–**10***(general method).* To a solution of 0.0029 mol of β-aminopropioamidoximes **1**–**5** in 20 mL of CHCl_3_, 0.0029 mol of DIPEA was added. The reaction mixture was cooled to −1 °C, and a solution of 0.0029 mol of 2-nitrobenzenesulfochloride in 2 mL of CHCl_3_ was added dropwise with stirring. The reaction mixture was then allowed to warm to r.t. (or up to the b.p. of CHCl_3_) and stirred until the completion of the reaction. The progress of the reaction was monitored by TLC. The formed white precipitates of the products were filtered off and recrystallized from 2-PrOH.

*2-Amino-1,5-diazaspiro[4.5]dec-1-en-5-ammonium 2-nitrobenzenesulfonate* (**11**). The reaction mixture consisting of 0.5 g (0.0029 mol) of compound **1** in 20 mL of CHCl_3_ and 0.38 g (0.0029 mol) of DIPEA was cooled to −1 °C. Then, 0.64 g (0.0029 mol) of 2-nitrobenzenesulfochloride was added dropwise. When the reaction mixture was kept at r.t. for 35 h, 0.80 g (77%) of white solid **11** was obtained (when the reaction mixture was kept at CHCl_3_ b.p. for 29 h, 0.78 g (75%) of white solid **11** was obtained); m.p. 153 °C, *R*_f_ 0.05. IR (KBr, cm^−1^): 1657 (C=N), 1593 (C=C), 1221, 1232 (SO_2_ as) and 1024 (SO_2_ sy), 1541 (NO_2_ as) and 1377 (NO_2_ sy), 3399 [N(-H)_2_], 2949 (C_sp3_−H), 3204, 3398 (C_sp2_−H). ^1^H-NMR (500 MHz, DMSO-*d*_6_): 3.05 (t, J = 7.95 Hz, 2H, α-CH_2_), 3.77 (t, J = 7.95 Hz, 2H, β-CH_2_), 1.54m, 1.71m, 1.84m, [6H, (CH_2_)_3_], 3.35 [m, 4H, N(+)(CH_2_)_2_], 7.20d, 7.46−7.81 (m, 4H, C_(sp2)_H). ^13^C-NMR (126 MHz, DMSO-*d*_6_): 21.0, 21.9(2C), 31.6, 60.7, 64.3, 122.0, 129.5, 130.5, 131.3, 140.0, 148.3, 168.6. Anal. Calcd for C_14_H_20_N_4_O_5_S (356.40): C, 47.18; H, 5.66. Found: C, 47.57; H, 5.33.

*2-Amino-8-oxa-1,5-diazaspiro[4.5]dec-1-ene-5-ammonium 2-nitrobenzenesulfonate* (**12**). The reaction mixture consisting of 0.5 g (0.0029 mol) of compound **2** in 20 mL of CHCl_3_ and 0.38 g (0.0029 mol) of DIPEA was cooled to −1 °C. Then, 0.64 g (0.0029 mol) of 2-nitrobenzenesulfochloride was added dropwise. When the reaction mixture was kept at r.t. for 25 h, 0.97 g (93%) of white solid **12** was obtained (when the reaction mixture was kept at CHCl_3_ b.p. for 24 h, 0.67 g (64%) of white solid **12** was obtained); m.p. 148 °C, *R*_f_ 0.01. IR (KBr, cm^−1^): 1637 (C=N), 1593 (C=C), 1207, 1228 (SO_2_ as) and 1022 (SO_2_ sy), 1549 (NO_2_ as) and 1377 (NO_2_ sy), 3387 [N(-H)_2_], 2907 (C_sp3_−H), 3250, 3306, 3328 (C_sp2_−H). ^1^H-NMR (500 MHz, DMSO-*d*_6_): 3.09 (t, J = 7.95 Hz, 2H, α-CH_2_), 3.88 (m, 2H, β-CH_2_), 3.35 [m, 2H, O(CH_ax_)_2_] and 3.60 [m, 2H, O(CH_eq_)_2_], 3.88 [m, 4H, N(+)(CH_2_)_2_], 7.20 (s, 2H, NH_2_), 7.45−7.81 (m, 4H, C_(sp2)_H). ^13^C-NMR (126 MHz, DMSO-*d*_6_): 31.5, 62.2 (2C), 63.2, 122.0, 129.5, 130.6, 131.3, 140.0, 148.3, 169.2. Anal. Calcd for C_13_H_18_N_4_O_6_S (358.37): C, 43.57; H, 5.06. Found: C, 43.89; H, 5.47.

*2-Amino-8-thia-1,5-diazaspiro[4.5]dec-1-en-5-ammonium 2-nitrobenzenesulfonate* (**13a**) *and 2-Amino-8-thia-1,5-diazaspiro[4.5]dec-1-en-5-ium chloride hydrate* (**13b**). The reaction mixture consisting of 0.55 g (0.0029 mol) of compound **3** in 20 mL of CHCl_3_ and 0.38 g (0.0029 mol) of DIPEA was cooled to −1 °C. Then, 0.64 g (0.0029 mol) of 2-nitrobenzenesulfochloride was added dropwise. When the reaction mixture was kept at r.t. for 104 h, the white precipitate of the mixture of products **13a** and **13b** was obtained. After recrystallization, 0.16 g (25%) of **13b** was obtained. After the evaporation of the filtrate from the recrystallization to ½ volume. 0.27 g (25%) of 2-nitrobenzenesulfonate **13a** was formed. When the reaction mixture was kept at CHCl_3_ b.p. for 24 h, only 0.37 g (56%) of white solid **13b** was obtained.

**13a:** m.p. 138−140 °C, *R*_f_ 0.08. IR (KBr, cm^−1^): 1647 (C=N), 1604 (C=C), 1207, 1215 (SO_2_ as) and 1024 (SO_2_ sy), 1545 (NO_2_ as) and 1373 (NO_2_ sy), 3497 [N(-H)_2_], 2936, 2964 (C_sp3_−H), 3152, 3258, 3302 (C_sp2_−H). ^1^H-NMR (500 MHz, DMSO-*d*_6_): 3.10 (t, J = 7.0 Hz, 2H, α-CH_2_), 3.83 (t, J = 7.95 Hz, 2H, β-CH_2_), 3.10 [m, 2H, S(CH_ax_)_2_] and 3.55 [m, 2H, S(CH_eq_)_2_], 3.10 [m, 2H, N(+)(CH_ax_)_2_] and 3.68 [m, 2H, N(+)(CH_eq_)_2_], 7.26 (s, 2H, NH_2_), 7.47−7.81 (m, 4H, C_(sp2)_H). ^13^C-NMR (126 MHz, DMSO-*d*_6_): 23.2, 31.5, 62.6, 64.7, 122.9, 129.5, 130.6, 131.3, 139.7, 148.3, 169.1. Anal. Calcd for C_13_H_18_N_4_O_5_S_2_ (374.44): C, 41.70; H, 4.85. Found: C, 41.67; H, 4.46.

**13b:** m.p. > 280 °C, *R*_f_ 0.08. IR (KBr, cm^−1^): 1659 (C=N); 1612 [H−N; (H)_2_−O]; 670 [S−C]; 3135, 3230, 3380, 3384 (H−O, H−N). ^1^H-NMR (500 MHz, DMSO-*d*_6_): 3.10 (t, J = 7.0 Hz, 2H, α-CH_2_), 3.85 (t, J = 7.95 Hz, 2H, β-CH_2_), 2.85 [m, 2H, S(CH_ax_)_2_] and 3.55 [m, 2H, S(CH_eq_)_2_], 3.10 [m, 2H, N(+)(CH_ax_)_2_] and 3.68 [m, 2H, N(+)(CH_eq_)_2_], 7.26 (s, 2H, NH_2_). ^13^C-NMR (126 MHz, DMSO-*d*_6_): 23.2, 31.5, 62.6, 64.7, 169.1. Anal. Calcd for C_7_H_16_ClN_3_OS (225.74): C, 37.24; H, 7.14. Found: C, 37.52; H, 7.48.

*2-Amino-8-phenyl-1,5,8-triazaspiro[4.5]dec-1-en-5-ammonium 2-nitrobenzenesulfonate* (**14**). The reaction mixture consisting of 0.72 g (0.0029 mol) of compound **4** in 20 mL of CHCl_3_ and 0.38 g (0.0029 mol) of DIPEA was cooled to −1 °C. Then, 0.64 g (0.0029 mol) of 2-nitrobenzenesulfochloride was added dropwise. When the reaction mixture was kept at r.t. for 34 h, 0.99 g (79%) of white solid **14** was obtained (when reaction mixture was kept at CHCl_3_ b.p. for 24 h 1.02 g (81%) white solid **14** was obtained); m.p. 185−187 °C, *R*_f_ 0.08. IR (KBr, cm^−1^): 1647 (C=N), 1593 (C=C), 1217, 1224 (SO_2_ as) and 1024 (SO_2_ sy), 1535 (NO_2_ as) and 1366 (NO_2_ sy), 3373 [N(-H)_2_], 2837 (C_sp3_−H), 3161, 3300 (C_sp2_−H). ^1^H-NMR (500 MHz, DMSO-*d*_6_): 3.12 (t, J = 7.91 Hz, 2H, α-CH_2_), 3.91 (t, J = 7.91 Hz, 2H, β-CH_2_), 3.40 [m, 2H, N(CH_ax_)_2_], 3.71 [m, 2H, N(CH_eq_)_2_], 3.54 [m, 4H, N(+)(CH_2_)_2_], 6.82−7.81 (m, 11H, NH_2_, C_(sp2)_H). ^13^C-NMR (126 MHz, DMSO-*d*_6_): 31.6, 44.6, 61.5, 62.9, 116.4, 120.5, 122.9, 129.5, 129.7, 130.6, 131.3, 139.8, 148.3, 150.0, 169.2. Anal. Calcd for C_19_H_23_N_5_O_5_S (433.48): C, 52.64; H, 5.35. Found: C, 52.92; H, 5.63.

When reaction mixture was kept at r.t. for 34 h, 0.99 g (79%) white solid **14** was obtained [when reaction mixture was kept at CHCl_3_ b.p. for 24 h 1.02 g (81%) white solid **14** was obtained].

### 3.2. Screening

The in vitro antidiabetic activity of samples **6**–**14** was assessed by the degree of inhibition of α-amylase and α-glucosidase activity. Pure DMSO was used as a solvent. The final concentration of the sample substances was 10 mg/mL. A total of 100 µL of α-amylase or α-glucosidase (1 U/mL) and 200 µL of the test sample solution (10 mg/mL) were added to 500 µL of phosphate buffer (0.1 M; pH 6.8). The resulting mixture was incubated for 15 min at +37 °C, and 200 µL of P-NPG solution (5 mM) was added.

Then, the resulting mixture was incubated again at +37 °C for 20 min. The reaction was stopped by the addition of 500 μL of sodium carbonate (0.1 M). Since the samples showed too much absorbance at 405 nm, they were diluted 5 times with 5 mL of water and 1 mL of sodium carbonate solution (0.1 M). A solution of α-amylase or α-glucosidase (1 U/mL) was used as a blank. As a negative control, 200 μL of pure DMSO was used in triplicate. As a reference drug, acarbose was obtained at a concentration of 10.0 mg/mL (positive control).

Simultaneously, a negative control was placed without the addition of the test compounds. All the samples were examined in triplets. The inhibitory activity was expressed as a percentage (%) of the degree of inhibition of α-glucosidase, in comparison to the negative control.

### 3.3. Single-Crystal X-ray Diffraction

The X-ray diffraction data of **8**, **9**, and **11**–**14** were collected on a Bruker Apex II diffractometer (Bruker AXS, Inc., Madison, WI, USA) equipped with an Oxford Cryostream cooling unit and a graphite monochromated Mo anode (λ = 0.71073 Å). The intensities of the reflections for **10** were collected at 100 K at the “Belok” beamline of the Kurchatov Synchrotron Radiation Source (NRC “Kurchatov Institute”, Moscow, Russia), at the wavelength of 0.745 Å using a MAR CCD 165 detector. The image integration was performed using the iMosflm software [35]. The integrated intensities were empirically corrected for the absorption using the Scala program [36]. The crystal structures were solved using SHELXT [37] program and refined with SHELXL [38] using OLEX2 software [39]. The structures were refined by the full-matrix least-squares procedure against F^2^. Non-hydrogen atoms were refined anisotropically. The H(C) positions were calculated, the H(N) and H(O) atoms were located on difference Fourier maps and refined using the riding model. The details of the experiment and the crystal parameters are presented in Tables S1 and S2 (Electronic Supporting Information).

## 4. Conclusions

The set of the reaction products of β-aminopropioamidoximes nitrobenzenesulfochlorination depends on the structure of the initial substrates and temperature. β-Aminopropioamidoximes with six-membered heterocycles in the β-aminogroup produce good yields of 2-amino-1,5-diazaspiro[4.5]-dec-1-ene-5-ammonium nitrobenzenesulfonates at r.t. and CHCl_3_ b.p. An exception is the *ortho*-nitrobenzensulfochlorination of β-(thiomorpholin-1-yl)propioamidoxime, when the reaction is regioselective at r.t., as two products are formed: 2-amino-1,5-diazathiospiro[4.5]-dec-1-ene-5-ammonium *ortho*-nitrobezenesulfonate and chloride hydrate. Heating leads to a regiospecific course of the reaction with the formation of only chloride hydrate.

The *para*-Nitrobenzenesulfochlorination of β-(benzimidazol-1-yl)propioamidoxime produces the O-*para*-nitrobenzenesulfochlorination product. The reaction time when the reaction mixture is heated is reduced by 2–3 times. The in vitro screening of the library of nitrobenzenelsulfochlorination products for antidiabetic activity reveals two samples with high α-glucosidase activity exceeding the activity of the acarbose standard: products of *para*-nitrobenzeneulfochlorination of β-(thiomorpholin-1-yl)- and β-(benzimidazol-1-yl)propioamidoximes. The arsenal of the physicochemical and spectral methods made it possible to establish the structural features of the studied spiropyrazolinium organic salts. Thus, in DMSO-*d*_6_ solutions in the ^1^H NMR spectra, the slow inversion of six-membered nitrogen-containing β-heterocycles can be observed. These spiropyrazoline derivatives can be interesting objects in dynamic NMR spectroscopy, which would allow for the rotational barriers of six-membered heterocycles to be measured. It is assumed that the bulky substituted arylsulfonate anion anchors the spiroheterocycles in the most thermodynamically favorable chair-like position. According to X-ray diffraction data, the axial location of the N–N bond in the spiropyrazoline heterocycles is unambiguously determined. The NMR and XRD data demonstrate that two various conformations of spirocation are present both in solution and in solids. The cation can take part in different types of intermolecular interactions, depending on the conformation and the nature of the six-membered cycle.

## Figures and Tables

**Table 1:** Physicochemical data on the products as a result of the nitrobenzenesulfochlorination of β-aminopropioamidoximes (**6**–**14**).

Compd	Yield,%		Time, h		M.p., ^°^C	*R*_f_	Compd	Yield,%		Time, h		M.p., °C	*R*_f_
	r.t.	70 °C	r.t.	70 °C				r.t.	70 °C	r.t.	70 °C		
**6**	77	46	38	27	151	0.12	**11**	77	75	35	29	153	0.05
**7** [7]	70	74	120	24	187–188	0.10	**12**	93	64	25	24	148	0.01
**8**	68	47	84	21	230	0.10	**13a**	25	-	104	24	138–140	0.08
**9**	70	69	60	19	203	0.15	**13b** [26,27]	25	56	104	24	>280	0.08
**10**	82	36	80	36	158	0.75	**14**	79	81	34	24	185–187	0.08

**Table 2:** In vitro α-amylase and α-glucosidase activity of nitrobenzenesulfochlorination products of β-aminopropioamidoximes (**6**–**14**).

**Compd**	**6**	**7**	**8**	**9**	**10**	**Acarbose**
α-Amylase activity, %	46.0 ± 2.8	27.7 ± 1.9	43.9 ± 2.1	42.0 ± 2.3	44.1 ± 2.9	50.3 ± 1.1
α-Glucosidase activity, %	“0”	48.1 ± 22.2	67.1 ± 3.8	36.5 ± 13.2	61.0 ± 1.5	58.9 ± 1.8
**Compd**	**11**	**12**	**13a**	**13b**	**14**	**Acarbose**
α-Amylase activity, %	17.2 ± 1.2	9.6 ± 2.2	3.4 ± 1.1	8.4 ± 0.8	14.3 ± 4.1	34.6 ± 0.4
α-Glucosidase activity, %	“0”	8.7 ± 2.2	“0”	“0”	13.0 ± 2.0	60.7 ± 0.7

**Table 3:** Selected geometrical parameters of spirocations **6**–**9**, **11**–**14** (Å and °).

	6	7 [7]	8	9	11	12	13a	14
N1–N2	1.470(2)	1.466(2)	1.468(2)	1.468(4)	1.469(1)	1.473(2)	1.460(5)	1.468(1)
N1–C1	1.526(2)	1.514(3)	1.526(2)	1.530(4)	1.513(1)	1.520(2)	1.493(7)	1.518(1)
C1–C2	1.485(3)	1.495(3)	1.497(3)	1.518(4)	1.517(2)	1.513(2)	1.526(9)	1.526(2)
N2=C	1.298(2)	1.299(2)	1.300(2)	1.316(3)	1.311(1)	1.305(2)	1.289(6)	1.300(2)
X–C_Ar_	1.492(4)–1.529(4)	1.401(3)–1.406(3)	1.791(3)–1.796(3)	1.464(4)–1.467(4)	1.519(1)–1.522(1)	1.434(2)–1.439(2)	1.794(5)–1.799(5)	1.466(1)–1.473(2)
d(C...Pz) ^1^	0.302(4)	0.418(4)	0.267(3)	0.425(5)	0.457(1)	0.445(2)	0.450(7)	0.435(2)
C_Ph_–NO_2_	1.472(2)	1.470(2)	1.477(2)	1.474(4)	1.472(1)	1.474(2)	1.454(6)	1.474(2)
C_Ar_-X-C_Ar_	112.1(2)	109.7(2)	98.2(1)	111.0(2)	109.83(9)	110.21(9)	95.5(2)	108.43(9)
Ω ^2^	8.9(1)	6.6(1)	4.9(1)	6.8(1)	112.63(4)	109.74(5)	113.20(2)	125.73(5)

^1^ The deviation of the C(1) atom from the mean plane formed by N–N=C–C atoms in the 5-membered pyrazole ring. ^2^ The twist angle between the mean planes of the phenyl ring and the NO_2_ group for the anion.

## Data Availability

The data presented in this study are available in this article.

## Supplementary Materials

The following supporting information can be downloaded at: https://www.mdpi.com/article/10.3390/molecules27072181/s1, Figure S1: Asymmetric units of the X-rayed compounds in a representation of atoms with thermal ellipsoids (*p* = 50%); Table S1: Crystallographic data and the experimental details for compounds **6** and **8**–**10**; Table S2: Crystallographic data and the experimental details for compounds **11**–**14**; Compounds **6** and **8**–**14** are registered in CCDC with the numbers 2154973-2154980. Crystallographic information files are available from the Cambridge Crystallographic Data Center upon request (http://www.ccdc.cam.ac.uk/structures).

## Sample Availability

Samples of the compounds **6**–**14** are available from the authors.
