# Supplementary material for: Reaction Products of β-Aminopropioamidoximes Nitrobenzenesulfochlorination: Linear and Rearranged to Spiropyrazolinium Salts with Antidiabetic Activity
Source: Molecules. 2022 Mar 28;27(7):2181. doi: 10.3390/molecules27072181 (PMC9000269; doi:10.3390/molecules27072181)
Supplement: Supplementary file 1 [file molecules-27-02181-s001.zip › molecules-1636904-supplementary.pdf]

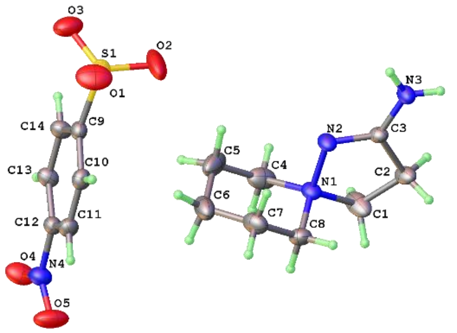

(**6**)

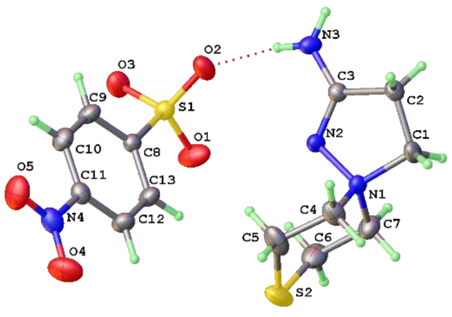

(**8**)

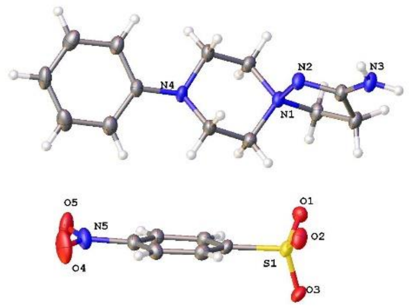

(**9**)

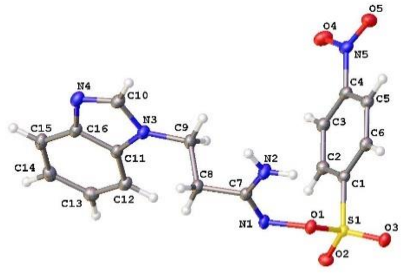

(**10**)

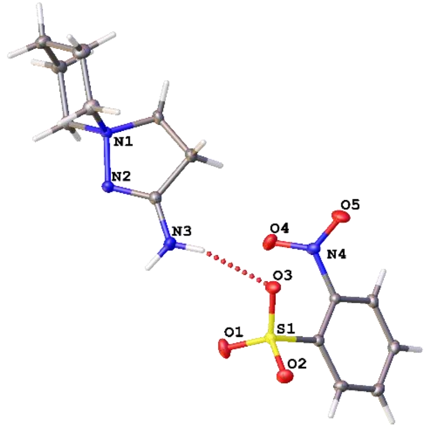

(**11**)

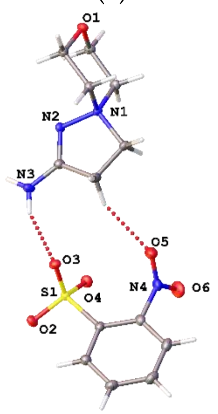

(**12**)

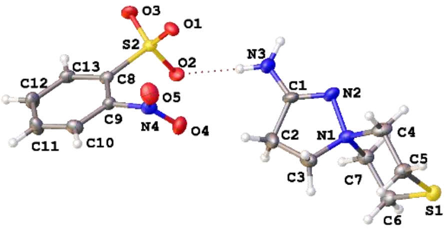

(**13a**)

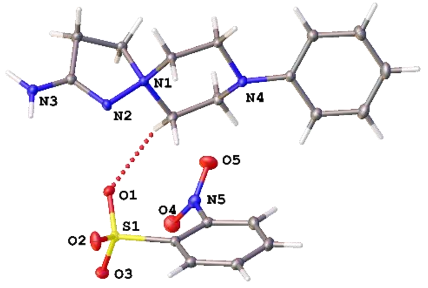

(**14**)

Figure S1. Asymmetric units of X-rayed compounds in representation of atoms with thermal ellipsoids (p = 50%).

Table S1. Crystallographic data and the experimental details for compounds **6**, **8**, **9**, **10**

Parameter	**6**	**8**	**9**	**10**
Empirical formula	$C_{14}H_{20}N_4O_5S$	$C_{13}H_{18}N_4O_5S_2$	$C_{19}H_{23}N_5O_5S$	$C_{16}H_{15}N_5O_5S$
Formula weight	356.40	374.43	433.48	389.39
Color, habit	Colorless, prism	Colorless, plate	Colorless, needle	White, prism
Crystal size (mm)	0.52 × 0.33 × 0.29	0.32 × 0.18 × 0.06	0.24 × 0.05 × 0.04	0.07 × 0.05 × 0.02
a (Å)	6.1065(5)	28.2148(16)	14.974(8)	8.7610(18)
b (Å)	10.5669(8)	6.1272(4)	8.465(5)	6.4250(13)
c (Å)	13.0230(10)	19.0811(12)	15.574(8)	29.525(6)
α (º)	98.148(3)	90	90	90
β (°)	100.441(3)	103.079(2)	96.772(14)	90.78(3)
γ (°)	97.976(3)	90	90	90
V (Å^3)	806.32(11)	3213.1(3)	1960.3(18)	1661.8(6)
Crystal system	Triclinic	Monoclinic	Monoclinic	Monoclinic
Space group, Z	$P\bar{1}$, 2	C 2/c, 8	P 2_1/n, 4	P 2_1/c, 4
D_{cal}(g cm^{-3})	1.468	1.548	1.469	1.556
μ (mm^{-1})	0.235	0.365	0.209	0.249
Reflections collected	25221	29230	21338	23047
Independent reflections (R_{int})	5594 (0.064)	4874 (0.072)	6178 (0.160)	4508 (0.028)
Obs.refl./restraints/ parameters	4421 / 45 / 217	3554 / 0 / 217	2583 / 0 / 272	4036 / 0 / 245
R,[a] % [F^2> 2σ(F^2)]	0.062	0.050	0.080	0.034
R_w,[b] % (F^2)	0.144	0.139	0.217	0.088
GOF [c]	0.974	1.02	0.93	0.971
F(000)	376	1568	912	808
CCDC	2154973	2154974	2153975	2153976

[a]$R = \Sigma||F_o|-|F_c||/\Sigma|F_o|$. [b]$R_w = [\Sigma(w(F_o^2 - F_c^2)^2)/\Sigma(w(F_o^2))]^{1/2}$. [c]$GOF = [\Sigma w(F_o^2 - F_c^2)^2/(N_{obs} - N_{param})]^{1/2}$

Table S2. Crystallographic data and the experimental details for compounds **11 – 14**.

Parameter	**11**	**12**	**13a**	**14**
Empirical formula	$C_{14}H_{20}N_4O_5S$	$C_{13}H_{18}N_4O_6S$	$C_{13}H_{18}N_4O_5S_2$	$C_{19}H_{23}N_5O_5S$
Formula weight	356.40	358.37	374.43	433.48
Color, habit	Colorless, prism	Colorless, plate	Colorless, prism	Colorless, prism
Crystal size (mm)	0.36 × 0.29 × 0.25	0.29 × 0.24 × 0.06	0.21 × 0.17 × 0.12	0.31 × 0.28 × 0.19
a (Å)	7.4003(2)	7.8995(3)	7.6475(5)	7.8520(3)
b (Å)	24.4135(6)	24.7412(12)	25.4264(15)	19.6923(6)
c (Å)	9.5475(2)	8.2533(3)	9.0081(6)	25.6870(9)
α (º)	90	90	90	90
β (°)	109.269(1)	105.063(1)	111.750(4)	90
γ (°)	90	90	90	90
V (Å^3)	1628.29(7)	1557.63(11)	1626.91(18)	3971.8(2)
Crystal system	Monoclinic	Monoclinic	Monoclinic	Orthorhombic
Space group, Z	P 2_1/n, 4	P 2_1/n, 4	P 2_1/n, 4	P b c a, 8
D_{cal}(g cm^{-3})	1.454	1.528	1.529	1.450
μ (mm^{-1})	0.232	0.248	0.360	0.207
Reflections collected	25374	15805	15047	56783
Independent reflections (R_{int})	6839 (0.052)	5605 (0.039)	4956 (0.119)	6073 (0.053)
Obs.refl./restraints/ parameters	5579 / 0 / 217	4343 / 0 / 217	2848 / 0 / 217	5241 / 0 / 271
R,[a] % [F^2> 2σ(F^2)]	0.039	0.040	0.093	0.037
R_w,[b] % (F^2)	0.099	0.095	0.180	0.095
GOF [c]	1.00	1.00	1.07	1.00
F(000)	752	752	784	1824
CCDC	2154977	2154978	2154979	2154980

[a]$R = \Sigma||F_o| - |F_c||/\Sigma|F_o|$. [b]$R_w = [\Sigma(w(F_o^2 - F_c^2)^2)/\Sigma(w(F_o^2))]^{1/2}$. [c]$GOF = [\Sigma w(F_o^2 - F_c^2)^2/(N_{obs} - N_{param})]^{1/2}$
